# Current progress and open challenges for applying tyrosine kinase inhibitors in osteosarcoma

**DOI:** 10.1038/s41420-022-01252-6

**Published:** 2022-12-12

**Authors:** Chenglong Chen, Qianyu Shi, Jiuhui Xu, Tingting Ren, Yi Huang, Wei Guo

**Affiliations:** 1 Department of Orthopedics, Beijing Jishuitan Hospital, Beijing, People’s Republic of China; 2 Beijing Key Laboratory of Musculoskeletal Tumor, Peking University People’s Hospital, Beijing, People’s Republic of China; 3 Musculoskeletal Tumor Center, Peking University People’s Hospital, Beijing, People’s Republic of China

**Keywords:** Bone cancer, Tumour angiogenesis

## Abstract

Osteosarcoma (OS) is a mesenchymal-origin tumor that constitutes the most common primary malignant bone tumor. The survival rate of the patients has significantly improved since the introduction of neoadjuvant chemotherapy and extensive resection, but it has stagnated in recent 40 years. Tyrosine kinase inhibitors (TKIs) have played a key part in the treatment of malignant tumors. In advanced OS, TKIs including anlotinib, apatinib, sorafenib, etc. have significantly improved the progression-free survival of patients, while the overall survival remains unchanged. The main reason is the rapid and inevitable progress of acquired drug resistance of OS. However, as the application of TKIs in OS and other tumors is still in the exploratory phase, its drug resistance mechanism and corresponding solutions are rarely reported. Hence, in this review, we summarize knowledge of the applications of TKIs, the mechanism of TKIs resistance, and the attempts to overcome TKIs resistance in OS, which are the three potentially novel insights of TKIs in OS. Because most evidence is derived from studies using animal and cell models, we also reviewed clinical trials and related bioinformatics data available in public databases, which partially improved our understanding of TKIs applications.

## Facts

- Angiogenesis is a common and major carcinogenic pathway involved in OS development, and TKIs are targeted drugs that specifically inhibit protein tyrosine kinases.
- TKIs, such as Apatinib and Anlotinib, play an eminent role in the treatment of advanced OS, prolonging the disease-free survival of the patients.
- The application of TKIs did not reach a prespecified goal of overall patient survival in the patients due to the rapid and inevitable progress of acquired drug resistance.
- In most clinical studies the combination therapies were superior to the TKI alone in treating advanced OS patients.

## Open questions

- What is the main cause of the resistance to TKIs in OS patients?
- What combination treatment options can maximize overall patient survival while minimizing side effects?
- What are the new and effective targets for TKIs in OS?

## Introduction

OS is the most common primary bone malignancy in children and adolescents, comprising nearly 56% of bone and soft tissue sarcoma [1]. OS most frequently affects the distal femur and 30–50% of OS patients have distant metastasis mainly lung metastasis because of its excessive aggressiveness. Since the 1970s, the application of combinational treatment including surgical resection, neoadjuvant chemotherapy, and post-surgical chemotherapy has increased the 5-year survival rate of OS from 17% to 60–75%. However, such data in patients with lung metastasis drops to only 20–30%, making lung metastasis the major reason for the poor prognosis of OS patients [2–4]. To retard OS progression as well as lung metastasis and ameliorate a patient’s prognosis, novel therapeutic strategies, such as immunotherapy, targeted therapy, and gene therapy have raised accumulating attention in recent years.

Angiogenesis exerts a pivotal role in the tumor progression of many solid tumor types including OS [4, 5]. Recently, small molecules targeting angiogenesis represented by small molecule inhibitors targeting tyrosine kinases have been widely applied in tumor-targeted therapy [3]. Apatinib, a TKI, has diminished tumor burden and improved patient progression-free survival substantially in advanced OS patients that are insensitive to chemotherapy [6]. Nevertheless, such targeting medicines still have several issues that need to be solved during clinical application in OS treatment, such as adverse reactions, selection of tumor targets, the rapid development of drug resistance, unimproved overall survival rate, etc. Therefore, this study focuses on the most important issues in TKI application. Here, we provide a summary of TKI clinical application in OS treatment as well as research progress and discuss its underlying mechanism and prospects.

## Progress of TKI application in OS treatment

### Protein tyrosine kinases (PTK) and their inhibitors (TKI)

The systematic treatment comprising radical surgery and combination chemotherapy has promoted patients’ prognosis to a large extent and become the standard therapy of OS since the 1970s. However, therapeutic strategies are still limited to chemotherapy combined with surgical resection of primary and metastatic tumor lesions for recurrent or metastatic OS patients, with a long-term survival rate of only 20%.

The tumorigenesis and tumor progress derives from a series of multi-factor interactions, which contain aberrant regulation of polygenes, dysregulation of metabolic enzyme expression, genetic factors, etc. [7]. Protein tyrosine kinases (PTK) are crucial for the functional regulation of normal cells within numerous signaling factors and their abnormal activation and dysregulation can cause alterations in downstream signaling that is related to tumorigenesis [7, 8]. PTK exerts its role by catalyzing the transfer of the phosphate group in adenosine triphosphate (ATP) to the hydroxyl group of the corresponding functional tyrosine residue, leading to its phosphorylation and triggering the activation of downstream cascades [9, 10]. Based on its structure, PTK can be divided into receptor tyrosine kinase (RTK) and non-receptor tyrosine kinase (NRTK). There are 58 kinds of RTK from 20 different subfamilies and 32 kinds of NRTK from 10 different subfamilies within the currently known 90 types of PTK [8].

**Fig. 1:**
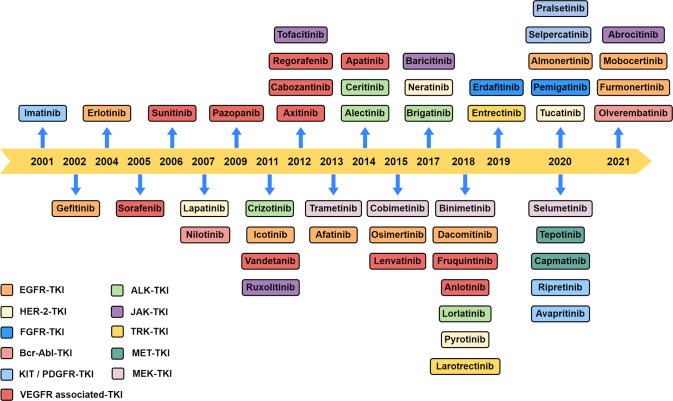
The main TKI drugs on the market in recent 20 years. The main TKIs approved in chronological order by China’s National Medical Products Administration, Japan’s Ministry of Health, Labour and Welfare and the US Food and Drug Administration in recent years.

Persistent activation of PTK due to mutation, translocation, or amplification is associated with tumor progression, invasion, and metastasis. Meanwhile, wild-type PTK can also participate in the initiation of tumor-related pathways as a key factor. Thus, making PTK become the major target of novel anti-tumor medicine research currently [11]. Since Food and Drug Administration (FDA) approved Imatinib as a second-line treatment of chronic myeloid leukemia in 2001, several potent well-tolerated drugs targeting PTK (including EGFR, ALK, ROS1, HER2, NTRK, VEGFR, RE, MET, MEK, FGFR, PDG, and KIT) have proven to play a gigantic role in tumor treatment [12]. These drugs called tyrosine kinase inhibitors (TKIs) exert tumoricidal effects through competitive inhibition of the corresponding kinase. Here we summarized the main TKI drugs in recent years (see Fig. 1).

**Fig. 2:**
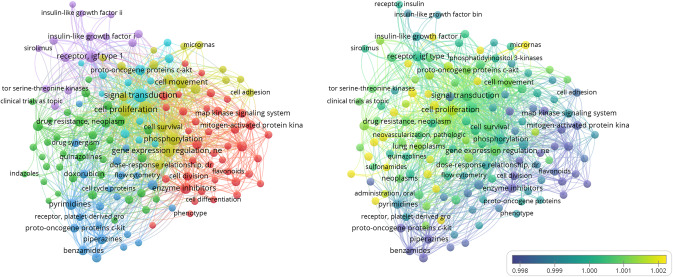
PubMed database was used to search literature related to OS and TKIs from 2000 to now, and the visual image of keyword distribution frequency was drawn, which showed that studies on TKIs in OS mostly focused on signal transduction, which was consistent with the action mechanism of TKIs. Keywords with the highest correlation included imatinib, IGF-1R, platelet-derived growth factor (PDGF), PI3K inhibitors, MAPK signaling pathway, etc.

Competitive inhibitors of ATP are typically a group of structurally related kinases such as pan-kinase inhibitors instead of selectively inhibiting only one kinase since the catalysis of PTK relies on the phosphate group of ATP as a substrate for all kinases, and the amino acid residues that form the ATP-binding site are generally highly conserved across all protein kinase families. In this process, to avoid the side effects caused by inhibiting other kinases, TKIs are usually designed as highly selective for one single kinase. Besides TKIs with one single target, there are many TKIs selective for multiple targets, for example, drugs targeting vascular endothelial growth factor receptor (VEGFR) [13]. However, many highly selective inhibitors were found to have inhibitory effects kinases in subsequent research and clinical practices. Many TKIs turn out to be multi-kinase inhibitors because of which they exert potent tumoricidal effects while causing several adverse reactions [14]. We searched the keywords in databases to perform a co-occurrence analysis of OS and TKIs. Figure 2 shows the results and indicates that checkpoint blockades such as imatinib, insulin-like growth factor 1 receptor (IGF-1R), PI3K inhibitors have been hot spots in recent years.

### Tyrosine kinase receptor-related targets and their inhibitors

#### Epidermal growth factor receptor (EGFR/erbB1)

As a member of the Erb family, EGFR is found to have aberrant activation in multiple solid tumor types, including non-small cell lung cancer, colorectal adenocarcinoma, head and neck squamous cell carcinoma, glioblastoma, breast cancer, etc. EGFR mutations are present in 2.8% of all cancers, the elevation of EGFR or its ligands such as tumor necrosis factor (TNF) leads to the initiation of downstream signaling that is related to tumorigenesis. A previous study demonstrated that high expression of EGFR could diminish OS sensitivity to chemotherapy and facilitate its drug resistance, while the application of anti-EGFR monoclonal antibody could enhance the tumoricidal effects of NK cells to OS [15]. HER-2 (erbB2) is a gene located on the long arm (17q12) of chromosome 17 that encodes a transmembrane protein with a similar structure to EGFR. HER-2 protein is a heterodimer of EGFR and forms the most active form of EGFR with it, and EGFR and HER-2 are the main therapeutic targets for patients with non-small cell lung cancer and breast or gastroesophageal cancer, respectively [16]. Some studies have suggested that high expression of HER-2 has a positive correlation to OS lung metastasis and recurrence [17, 18]. However, there are studies indicating that HER-2 was expressed in OS with a low level and the level of its expression was not associated with the pathological classification, tumor grade, preoperative chemotherapy response, and disease progression of OS [19, 20]. The inconsistent results from different studies indicate that there is relatively high heterogeneity in protein expression among patients with OS compared with other malignancies.

The therapeutic effects of monoclonal antibodies or inhibitors targeting EGFR and HER-2 have been widely verified in clinical trials focusing on non-small cell lung cancer and breast cancer, including Cetuximab, Amivantamab, Osimertinib, Poziotinib, Mobocertinib, Afatinib, and Tarloxotinib. Studies about the implementation of monoclonal antibodies or inhibitors targeting EGFR in OS are scarce and mostly focus on experiments on cell and animal levels. Nevertheless, a phase II clinical trial demonstrated that compared with OS patients with low HER-2 expression, a combination of Trastuzumab and Adriamycin could not promote the prognosis of OS patients with high HER-2 expression. The 30-month event-free survival rates of patients with low and high HER-2 expression were both 32%, and the overall survival rates were 50% and 59%, respectively [21].

#### Vascular endothelial growth factor receptor (VEGF/VEGFA)

**Fig. 3:**
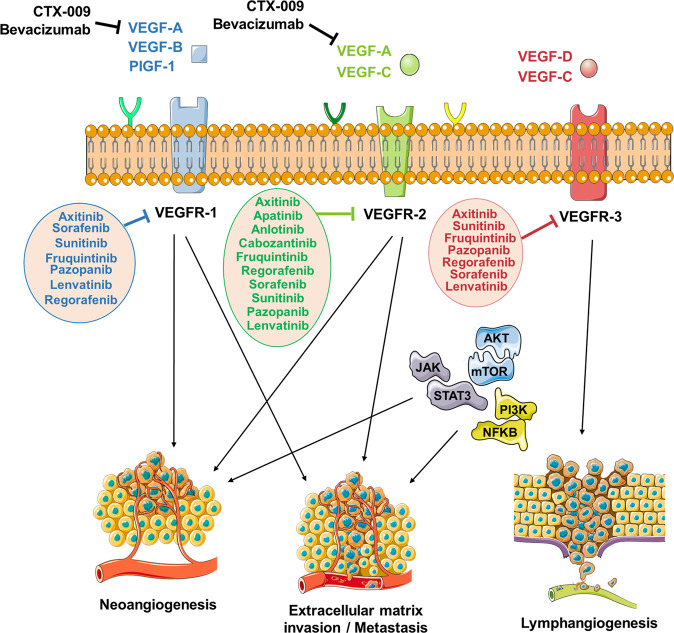
The main mechanism of TKIs developed with VEGF-related targets in OS. Some of the TKIs such as Bevacizumab and CTX-009 inhibit VEGF-A, -B, -C, or PIGF-1 to further reduce binding to specific receptors on the cell surface. Other TKIs such as Axitinib, Sorafenib, Apatinib, and Lencatinb inhibit VEGFR-1, -2, or -3 to restrain downstream pathways associated with neoangiogenesis, exracellular matrix, invasion, metastasis, and lymphangiogenesis.

VEGF mainly affects endothelial cells and is a key factor for angiogenesis. Up to 40% of OS patients exist platelet-derived growth factor (PDGFRA) or VEGF genome amplification and the high expression of VEGF is correlated to lung metastasis and poor prognosis of OS [22–24]. Our previous study suggested that the ligand of VEGF (VEGFR-2) was expressed in nearly 64.5% of OS patients and was associated with PD-L1 expression. Both VEGFR-2 and PD-L1 high expression indicated a poor prognosis [25]. Novel therapeutic strategies targeting VEGF-related targets are the focus of OS-targeted therapy research since OS has abnormal expression of VEGF and its ligands as well as abundant angiogenesis. Here we summarized the main mechanism of TKIs developed with VEGF-related targets in OS (see Fig. 3).

Many non-coding RNA is still able to modulate tumor progression via targeting VEGF in OS. For example, CircRNA-001621 can adsorb miR-578 that targets and inhibits VEGF transcriptome expression, thus boosting proliferation and metastasis of OS [26]. Our previous study indicated that miR-134 could impede proliferation and angiogenesis in OS via VEGF/VEGFR1 signaling [27]. TKI drugs targeting VEGFR-2 such as apatinib, anlotinib, and sunitinib can increase apoptosis and abrogate the proliferation, metastasis, and angiogenesis in OS [28–30]. Meanwhile, antiangiogenic drugs targeting VEGFR have received satisfying therapeutic effects in the clinical trials of advanced or recurrent OS patients. Our previous clinical trial showed the effectiveness of apatinib in the advanced OS that the 6-month event-free survival after receiving apatinib reached 36.5% in patients with advanced OS after chemotherapy failure [6]. In another two clinical trials of advanced OS patients, apatinib showed similar effectiveness and acceptable drug tolerance, which primed it for a highly targeting TKI drug in OS treatment [31, 32]. Similarly, the 6-month event-free survival after administration of Cabozantinib and Sorafenib in advanced OS patients achieved 33% and 45%, respectively, which were still less than the expected 50% [33, 34]. Therefore, utilizing combination therapy to promote curative effects and overcome the side effects of single drug administration as well as the most urgent problem that needs to be solved.

#### Insulin-like growth factor receptor

Insulin-like growth factor (IGF) is a cytokine with significant biochemical and physiological functions. IGF-1, IGF-2, and insulin can all activate and phosphorylate IGF1-R, which interacts with several intracellular second messenger pathways including Ras/Raf/MAPK and PI3K/AKT signaling pathways [35]. A preliminary study suggested that IGF and its signaling are vital for sarcoma development and progression. For example, IGF-1R expression in synovial sarcoma is related to its invasiveness and lung metastasis, while using IGF-1R antibody or knocking down its gene expression leads to abundant cell apoptosis and inhibition of tumor growth and lung metastasis [36].

The expression level of transcriptome and protein of IGF-1R is significantly higher than in normal bone tissue in OS lesions, while IGF-1R protein expression correlates to the stages and distal metastasis closely in OS patients [37]. High expression of IGF-1R indicates a poor prognosis and the level of IGF-1R expression is an independent prognostic indicator in OS patients. Inhibition of IGF-1R causes abrogation of the adhesion, migration, invasion, and metastasis of OS [37]. One study focusing on Ewing sarcoma demonstrated that combined administration of small molecule inhibitors of IGF-1R (Linsitinib and Trabectedin) could enhance the antitumor effects of trabectedin by suppressing IGF signaling [38]. IIGF-1R participates in the drug resistance of multiple antitumor medicines and inhibition of IGF-1R signaling and expression could reverse tumor resistance to chemotherapy drugs. Linsitinib, Robatumumab, and figitumumab have shown promising antitumor effects and are well-tolerated and dated in clinical trials of Ewing sarcoma, while their therapeutic effects on OS need further research [39–41].

#### Fibroblast growth factor receptor (FGFR)

Fibroblast growth factor receptor (FGFR) comprises four subtypes (FGFR-1/2/3/4) and the abnormal activation of FGFR leads to dysregulation of cell proliferation and suppression of cell apoptosis that may cause tumorigenesis. Studies showed that FGFR-1 participated in the regulation of c-Fos/AP-1 signaling, and the FGFR-1 transcriptome and protein expression level were upregulated in osteoblasts and chondroblasts that were induced to overexpress c-Fos in vitro, which caused the persistent activation of mitogen-activated protein kinase (MAPK) thus promoting OS growth and lung metastasis while administrating FGFR-1 inhibitors could diminish the number of lung metastasis lesions [42]. Simon M. Cool et al. induced tumor cell apoptosis via a kind of anti-FGFR-1-neutralizing serum that could suppress the combination of heparin and FGFR-1 and block the interaction of FGF-2 and FGF-R1 as well as the kinase activity of FGF-R1 [43]. In OS, FGF-R1 is an oncogene whose mutation is deemed as a latent biomarker while its inhibitor Infigratinib is thought to be a promising drug for OS patients with FGFR mutation [44]. Another study suggested that the administration of PLA nanoparticles or liposomes as a carrier of Ponatinib and Nintedanib to OS tumor cells could enhance their antitumor efficacy and lower their side effects at the same time [45]. Similarly, using biomimetic lipid nanoparticles as a carrier of Ponatinib could decrease the side effects and dampened efficacy caused by off-target effects of Ponatinib, thus inhibiting mouse OS cell proliferation effectively [46]. However, studies focusing on FGFR application in OS were mostly staying the cell and animal experiments stage, and the therapeutic effects of other MKI drugs targeting FGFR, such as Erdafitinib and Sulfatinib in OS still need further research.

#### Platelet-derived growth factor receptor (PDGFR)

Platelet-derived growth factor receptor (PDGFR) and its ligand (PDGF-A, PDGF-B, PDGF-C, and PDGF-D) are responsible for modulating mesenchymal cell (such as fibroblasts and pericytes) functions [47]. Such cells are the main components of tumor stroma and can influence the growth, metastasis, and sensitivity to the drug of the tumor. At least three different pathways exist by which PDGFR signal participate in tumor development and progression: (i) facilitating tumor cell proliferation directly through autocrine; (ii) stimulating tumor stromal cells via paracrine thus enhancing angiopoiesis to overcome hypoxia in the tumor microenvironment; (iii) modifying the fluid pressure of tumor stroma to control the influx and outflux of drugs thus diminishing the antitumor effects of drugs [48]. The expression of PDGF and its receptor PDGFR is generally high in OS, although their expression is heterogenetic (4–90%). The correlation between high expression of PDGF and poor prognosis of OS seems to be generally accepted. Studies suggested that OS patients with high expression of PDGF-A had a significantly lower 5-year disease-free survival rate compared with patients with low expression of PDGF-A (21.22% vs. 42.72%) [49]. Inhibitors targeting PDGFR (such as imatinib, sunitinib, sorafenib, and pazopanib) have achieved satisfying therapeutic effects in clinical trials of different tumor types. The antitumor effects of most TKIs (except for imatinib) to OS are attributed to their antiangiogenic effect other than the anti-PDGF effect.

The therapeutic effects of imatinib and inhibitor of PDGF-Rα (Olaratumab) were not satisfying even in patients with relatively high expression of PDGF/PDGFR in clinical trials of OS and other soft tissue sarcomas, indicating that single administration of these medicines is not a viable therapeutic strategy in patients with bone and soft tissue sarcoma. However, the combination of Olaratumab and Adriamycin could boost the therapeutic effects effectively as well as decrease the side effects. Combinational administration could reduce the risk of death by 48% in sarcoma patients compared to single-drug administration [50]. The effectiveness of combinational administration of PDGF/PDGFR inhibition was edified in non-small cell lung cancer, may which can be attributed to the anti-hypoxia function of PDGF/PDGFR inhibitors in the tumor microenvironment. Such function is especially obvious in sarcomas and the sensitivity of tumor and stromal cells to antitumor decatenating since correction of the hypoxic tumor environment. Therefore, the combinational administration of chemotherapy and other antitumor medicines is supposed to be the future direction for TKIs that achieve limited therapeutic effects was administrated solely.

#### Hepatocyte growth factor receptor

c-Met is the only receptor of hepatocyte growth factor (HGF) currently and is a highly binding cell membrane surface receptor tyrosine kinase that is encoded by proto-oncogene Met. The activation of c-Met leads to mediators of downstream signaling pathway activation (such as MAPK), which further initiates signaling pathways including Ras/MAPK, PI3K/AKT, and JAK/STA signaling that are capable of regulating the cell proliferation, migration, and apoptosis. Aberrant expression of c-Met causes alteration of cell survival and invasiveness and stimulation of angiogenesis, which promotes tumor growth and metastasis and induces resistance to antitumor agents [51]. The study indicated that the acquired resistance caused by the administration of sorafenib in hepatocellular carcinoma correlated to the immune evasion attributed to the high expression of PD-L1. While c-Met is responsible for modulating PD-L1 expression, MAPK signaling pathway, and downstream NF-κBp65 transcription factor are able to interact with the proximal region of PD-L1 promoter to facilitate PD-L1 expression synergistically [52].

The study showed that combinational administration of Rilotumumab targeting HGF and Car-T cell therapy could significantly suppress Ewing sarcoma growth, and the use of Rilotumumab could increase antitumor effect via facilitating leukocyte infiltration surrounding tumor [53]. As a multitarget MKI, Cabozantinib targets c-Met, and VEGFR-2 and achieves promising therapeutic effects in advanced sarcoma including OS and Ewing sarcoma. 87% of patients with Ewing sarcoma and 93% of patients with OS received evaluable efficacy and the 6-month progression-free survival rate of advanced OS patients was nearly 33% [34]. The study suggested that Cabozantinib inhibited tumor cells and altered the tumor microenvironment by suppressing ERK and AKT signaling pathways, which caused augmentation of tumor apoptosis and antitumor effect based on immune response in vivo [54]. The selection of different RTK inhibitors should be based on their target expression among different tumor types and different patients since there are numerous kinds of RTK inhibitors. Administration of RTK inhibitors combined with chemotherapy and immune checkpoint inhibitors may truly exert their anti-tumor efficacy.

### Non-receptor tyrosine kinase (NRTK)

Located in the cytoplasm and nucleus, NRTK initiates downstream signaling pathways (PI3K/AKT, JAK/STAT, and Ras/Raf/MAPK) through tyrosine phosphorylation of extracellular signals (from T lymphocyte receptors, B lymphocyte receptor, and immunoglobulin receptors) to form signal transduction complex, thus regulating cell growth and differentiation while aberrant NRTK activation leads to tumorigenesis [55]. Abnormal signal transduction of NRTK was also found in OS, which inhibited OS cell growth, invasion, and migration and augmented apoptosis by regulating the SRC/STAT3/BCL-2 signaling pathway [56]. PI3K/AKT signaling pathway was up-regulated in OS leading to facilitating OS proliferation directly and increasing its resistance to antitumor drugs including chemotherapy medicine [57]. Activated by FGF2 (a kind of RTK), PI3K/AKT signaling pathway could modify non-coding RNA targeting FGF2 to indirectly regulate OS growth and metastasis [58]. Currently, PI3K inhibitors are still in the stage of clinical trials in the treatment of OS, clinical trials containing immune checkpoint inhibitors (for example, anti-PD1antibodiesy) that could be administrated cooperatively are also in progress. Macrophage migration inhibitor (MIF) can activate RAS/MAPK signaling pathway time- and dose-dependently in OS, thus promoting tumor growth and lung metastasis. Inhibition of MIF leads to an increase in the sensitivity of OS cells to cisplatin and doxorubicin, which prime immune therapy targeting MIF to block the RAS/MAPK signaling way a viable therapeutic strategy for OS [59]. As the first discovered oncogene, Src kinase is activated aberrantly in several tumor types including breast cancer. Previous studies demonstrated that Src kinase was the center of signal transduction and could function as a multifunctional intracellular tyrosine kinase to coordinate cell reactions to external stimuli. More importantly, as the ligand of many growth factor receptors (including EGFR, PDGFR, and FGFR), Src kinase could expedite angiogenesis, tumor cell proliferation, invasion, and migration [60]. Since Src is a key signaling module in the cell, anti-OS therapy targeting Src kinase involves multiple tumor-related signaling pathways (such as PI3K/Akt/mTOR, ERK/c-MYC, and STAT3/BCL-2) with a complex mechanism and interaction. Therefore, it is predictable that agents targeting Src kinase will achieve various reactions among different patients while there is still a great possibility that implementing combinational administration to increase OS patients’ prognosis [56, 61, 62].

## Resistance of tyrosine kinase inhibitors in tumors

**Fig. 4:**
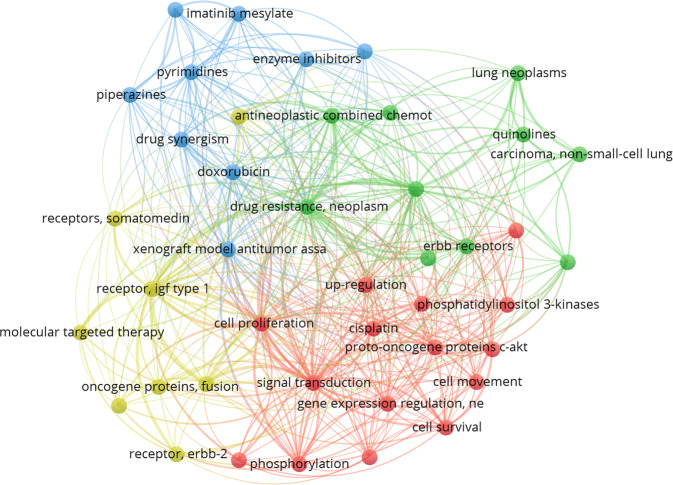
The research hotspot of TKIs resistance in OS. According to the results of visual analysis, the distribution frequency of keywords of drug resistance-related studies of all TKIs in OS was shown in the figure, indicating that the research focuses on IGF-1R, ErbB receptor, AKT, and PI3K pathway.

Most TKIs are still early stages and have limited application in the treatment of most tumor types. Thus, the mechanism of tumor resistance to TKIs is not clear currently and needs further research. TKIs are mostly administrated in advanced malignant tumors (for example advanced and recurrent OS after chemotherapy failure) with a satisfying therapeutic efficacy at first and could increase the disease-free survival of patients significantly. Nevertheless, drug resistance and side effects appear after persistent administration [63]. The occurrence of drug resistance makes the selection range of effective therapeutic strategies narrow, which further results in the survival of OS patients being stagnant and makes the solution of drug resistance of TKIs a vital issue to improve the survival rate of malignant tumors. Through visual analysis, we summarized the most frequent keywords of TKI resistance mechanism in OS (see Fig. 4).

**Fig. 5:**
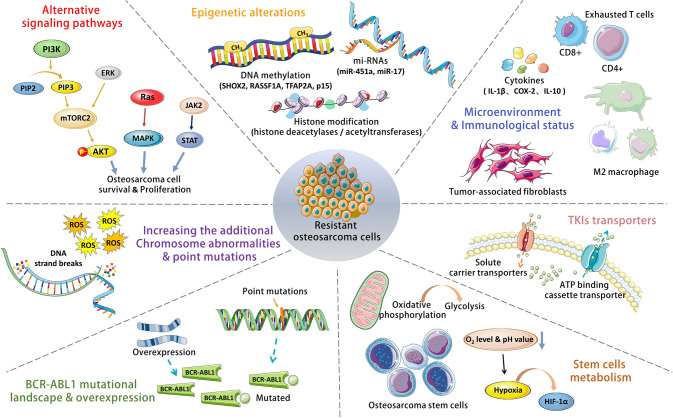
The common resistance mechanisms of TKIs in tumors. Dysregulation of tyrosine kinase activity is frequently found in tumors, of which the most clinically relevant and most studied is the abnormal expression of BCR-ABL1. BCR-ABL1 mutation was first identified as a point mutation caused by the substitution of isoleucine for threonine at position 315, which occurs most frequently in drug-resistant patients, and the effectiveness of TKIs is highly dependent on the interaction between BCR-ABL1 and THR, and the failure of TKI binding to the target kinase caused by the overexpression of BCR-ABL1 and fusion gene mutation may lead to drug resistance.

Generally, the mechanism of TKIs drug resistance including: amplification of target receptor expression, impaired TKI binding due to target receptor mutation, reduced TKI uptake by tumor cells through certain drug transport mechanisms, differentiation and proliferation of tumor stem cells, DNA repair and chromosomal variation, aberrant expression of non-coding RNA, abnormal regulation of immune cells caused by alterations in the tumor microenvironment, and activation of alternative pathways and downstream signaling in tumor cells [55]. We summarized the common resistance mechanisms of TKIs in tumors (see Fig. 5).

The discharge of tumor metabolites, exogenous substances, and drugs is conducted by ATP-binding cassette transporters, and such transporters’ overexpression leads to a decrease in intracellular drug concentration [64]. Such a process causes loss of efficacy of TKIs because of invalid intake or excretion by cells, which results in tumor cell resistance. Although relevant targets are combined effectively with TKIs, several signaling pathways (such as RAS/MAPK, PI3K/AKT/mTOR, EREG/EGFR, Src, and JAK/STAT signaling pathways) will be activated, compensate and replace the original kinase pathway. Activation of these pathways will generate downregulation of tumor cell apoptosis, an increase of autophagy, and a change of cell cycle that may counteract with TKIs antitumor effects. The regulation of transcriptome by non-coding RNA is of importance in OS drug resistance, metastasis, and recurrence. These non-coding RNAs utilize exosomes secreted by a tumor or tumor-related stromal cells and stem cells as a vehicle to transport into the extracellular environment. In addition, the hypoxia and pH imbalance in the tumor microenvironment, and abnormal expression of corresponding proteins on the surface of surrounding immune cells provide a permissive condition for drug resistance in OS. For example, M2 tumor-associated macrophages could regulate OS resistance to TKIs and chemotherapy via releasing cytokines including IL-1β, COX-2, and IL-10. And the overexpression of immune checkpoints such as PD-L1 on tumor cell membrane retards the antitumor immune response induced by medicines [65]. In summary, the process of drug resistance is complicated in tumors including OS, which involves changes in the small molecule drug target itself, and abnormalities in drug concentrations, immune cells, and other signaling pathways. Therefore, addressing drug resistance by a single approach may be negligible for improving patient prognosis, and combinational therapy with precise personalization, along with biomarkers that are capable of identifying drug responses is vital for more effective treatment options.

## Adverse effects of TKIs therapy in OS

TKI therapy in OS results in fewer adverse effects with lower severity compared with chemotherapy. However, reducing or suspending medication because of adverse effects caused by TKIs affects patient prognosis to a large extent. Meanwhile, the side effects of TKIs gained increasing attention since several kinds of TKIs treatment need lifelong medication. The common side reactions in TKI treatment of OS comprise oral mucositis, hand–foot syndrome, low platelet count, low white blood cell count, abnormal liver function, elevated blood bilirubin, hypertension, hypothyroidism, anorexia, abdominal pain, and diarrhea. Among them, the hand–foot syndrome is one of the most frequent adverse effects and the incidence of it achieves 37.8–48.8% in OS patients treated with apatinib, with a typical performance of painful and localized keratosis with erythema [66]. In clinical trials of pazopanib treatment of metastatic soft tissue sarcoma and regorafenib treatment of advanced OS, there were 18% and 51% of patients suffering from the hand–foot syndrome, respectively [67]. In these studies, 3–17% of patients presented with grade 3 severe hand–foot syndrome that interfered with life quality extremely. Such lesions commonly arise 45–80 days after medication and therefore, we should take positive interventions before skin lesions occur, including taking B vitamins orally, avoiding direct sunlight, using a moisturizer along with medication, and avoiding rubbing [68]. Similar to the hand–foot syndrome, oral mucositis occurs due to adverse effects caused by vasoconstriction by antiangiogenic drugs, with a general incidence rate of 11–38% for all types of TKIs. Most patients have oral mucositis 60 days after medication and local anesthetic or diphenhydramine hydrochloride could mitigate symptoms for patients with severe oral mucositis [69].

Hypertension is the common side effect of VEGFR inhibitors, with an incidence rate of 18.92% in a clinical trial of apatinib treatment of OS. Many studies indicated that hypertension is a clinical indicator of good prognosis [70]. However, the administration of angiogenesis inhibitors should be prudent for patients with hypertension or cerebrovascular diseases. The incidence rate of adverse reactions in the digestive system varies according to different drug types. In a clinical trial of renal carcinoma, the incidence rate of diarrhea caused by Cabozantinib was 50%. While the number turned to 27% and 26% in sorafenib and anlotinib treatment of OS, respectively [70–72]. Stomach ache usually arises along with diarrhea and lowers patient adherence to medication due to perturbation of water and electrolyte metabolism and weight loss. A patient’s condition can be effectively improved through dietary adjustment, drug withdrawal, or parenteral nutrition. In sum, determining whether the patient has a history of hypertension, cardiovascular and cerebrovascular diseases, and conducting a routine physical examination before TKIs administration could prevent severe side effects occurrence effectively.

## Application of combinational therapy of TKIs in OS

Multiple clinical trials showed that TKIs could prolong the progression-free survival of OS patients efficaciously while hardly influencing overall survival, which correlated to drug resistance and withdrawal because of adverse reactions. Finding a combinational therapeutic strategy based on TKIs is vital for overcoming tumor resistance, reducing adverse reactions, and promoting patient prognosis. Currently, several combinational strategies have been carried out in OS animal experiments and clinical trials and achieved certain therapeutic effects, such as a combination of TKIs with chemotherapy, immune checkpoint inhibitors, or other types of TKIs.

A retrospective clinical study suggested that a combination of apatinib and ifosfamide could decrease the tumor burden of advanced and recurrent OS patients, with a median progression-free survival of 11.4 months and median overall survival of 19.8 months. The combinational administration of apatinib and ifosfamide could retard tumor progression significantly compared with single-drug administration [73]. Our previous study indicated that apatinib inhibited OS invasiveness and metastasis through the downregulation of OS PD-L1 expression. Meanwhile, data from our clinical study showed that combinational treatment of apatinib and PD-L1 monoclonal antibody camrelizumab for advanced OS achieved longer disease-free survival compared with a single treatment of apatinib (with a 6-month progression-free survival rate of 50.9%) [25, 74]. The study demonstrated that triple therapy including Lenvatinib, etoposide, and ifosfamide could exert potent inhibition in OS cell lines and animal models [75]. Such triple therapy could increase patient 4-month progression-free survival from 32% to 51% compared with using Lenvatinib alone in a multicenter clinical trial in patients with recurrent OS, although the incidence of adverse reactions was not affected [76]. Similarly, combinational therapy comprising sorafenib and mTOR inhibitor everolimus could increase patient 6-month progression-free survival from 29% to 45% (although not reaching the expected 50% and above) compared with using sorafenib alone in recurrent and inoperable OS patients [33].

Thus far, we compared the clinical efficacy of combination therapy with that of TKIs alone in OS, and in most clinical studies the combination was superior to the TKI alone. Consequently, does it mean that TKI alone will need to be replaced by a combination in future clinical applications, and where is the way out for TKIs approach? The answers may emerge from nano-transporter-based and genetic therapy that can improve the efficacy of TKIs. Liao et al. used a nanomodified TKI to overcome the resistance in lung cancer [77], and this inspired us to use nano-formulations of TKIs to enhance the efficacy comparable to combination therapy but also to avoid the adverse effects associated with the combination therapy. These nano-formulations can be achieved by exosomes or extracellular vesicles as well as some novel nanoliposome materials. However, more trials are needed to verify and guarantee the true efficacy and histocompatibility of artificial nanoliposomes in the future. Correspondingly, copy number variations vary from patient to patient, the efficacy of gene therapy-assisted TKI drugs in OS needs to be adapted to the actual situation of different patients, and there is still a long way to go in terms of the operability of their clinical application. Therefore, improving the anti-drug resistance structure of the TKIs themselves and developing TKIs that are gnomically targeted to OS patients are the fundamental keys to addressing the resistance.

## Conclusions

Most combinational therapeutic strategies can achieve better therapeutic efficacy than single-drug administration in OS. However, problems still exist including inevitable adverse reactions and nearly unchanged overall survival in patients. Learning from experiences of combinational treatment for other malignant tumor types while conducting clinical trials, adopting combinational and sequential therapeutic strategies, and exploring the mechanism of drug resistance is essential for finding an effective combinational approach and developing next-generation TKI agents.
